# Prognostic value of cathepsin D in breast cancer

**DOI:** 10.1038/sj.bjc.6690032

**Published:** 1999-01

**Authors:** B R Westley, F E B May

**Affiliations:** Department of Pathology, Royal Victoria Infirmary, Newcastle upon Tyne, NE1 4LP, UK


British Journal of Cancer (1999) **79**(2), 189–190
© 1999 Cancer Research Campaign

Editorial

### Prognostic value of cathepsin D in breast cancer

**BR Westley and FEB May**

Department of Pathology, Royal Victoria Infirmary, Newcastle upon Tyne NE1 4LP, UK

The paper in this edition by John Foekens's group at the Rotterdam Cancer Institute (Foekens et al, 1998) represents something of a landmark in the assessment of cathepsin D as a prognostic marker in breast cancer. It demonstrates unequivocally that cathepsin D is an independent marker of poor prognosis in all groups of breast cancer patients.

Cathepsin D is an aspartyl protease that is normally localized within lysosomes and is involved in protein catabolism and tissue remodelling (Westley and May, 1996). For reasons that are not clear, but probably as a result of intracellular misrouting, this protease can be secreted. The secretion of cathepsin D is elevated in some cancer cells, and it has been suggested that the illicit secretion of a protease whose activity is normally restrained to the intracellular degradation of proteins may facilitate the invasion and spread of cancer cells.

Cathepsin D synthesis and secretion is regulated by oestrogen in oestrogen-responsive breast cancer cells (Westley and Rochefort, 1980); however, the reason why cathepsin D is regulated by oestrogen remains obscure. Given its role in protein catabolism, it may be involved in tissue involution in oestrogen-responsive tissues, and examples that immediately spring to mind are the involution of ductal and alveolar structures in the breast following the cessation of lactation and the post-partum involution of the uterus. The regulation of cathepsin D expression by oestrogen results from the interaction of the oestrogen receptor with oestrogen response elements in the cathepsin D promoter. The classical model of oestrogen action that involves the interaction of receptor homodimers with a palindromic oestrogen response element of the type found in the *Xenopus laevis* vitellogenin gene promoter does not seem to apply to cathepsin D.

Cathepsin D expression originally became of clinical interest in breast cancer as a potential marker of oestrogen responsiveness. It was hoped that it would prove more reliable than existing markers such as the oestrogen receptor; however, the promise of cathepsin D as a marker of oestrogen responsiveness has not been fulfilled. Interest in this protease has continued as a result of reports that it is a marker of poor prognosis in node-negative breast cancer patients (Spyratos et al, 1989; Foekens et al, 1996). The value of identifying markers of poor prognosis in node-negative breast cancer is that we need to be able to identify those women in this good-prognosis group who are at significant risk of relapse and who would be likely to benefit from a more aggressive treatment regimen.

Research into the prognostic value of cathepsin D has been facilitated by the availability of a radiometric immunoassay that has been validated by the EORTC Breast Cancer Cooperative Group (Benraad et al, 1992). Although this assay is expensive to perform, it measures the amount of cathepsin D in tumour cytosols prepared for oestrogen receptor analysis with impressively low inter-assay variation. It should be noted that at least three other methods have been used to measure cathepsin D expression in prognostic studies, namely Western transfer, immunohistochemistry and direct measurement of cathepsin D enzymatic activity. While the quality control inherent in the radiometric assay is of obvious benefit, pathology laboratories are increasingly called upon to measure the expression of prognostic factors and generally prefer immunohistochemical assays. These, although semiquantitative, are able to provide potentially valuable information on cellular localization. The development of a prognostically validated immunohistochemical assay for cathepsin D would undoubtedly bring cathepsin D closer to clinical relevance, in contrast to its widely regarded current status as a research curiosity.

The major issue addressed by the paper of Foekens et al (1998) is one that has been facing breast cancer patients, the clinicians treating them and the pharmaceutical company involved in producing the cathepsin D assay for more than 10 years, i.e. the usefulness of cathepsin D as a prognostic marker to identify women with a high risk of relapse. Evaluation of cathepsin D as a prognostic marker has been seriously hampered both by the numbers of patients included in the clinical studies (very few studies have involved more than 150 patients) and by the relatively short follow-up times (Westley and May, 1996). The study of Foekens et al (1998) is remarkable in that it involved the analysis of 2810 cytosolic extracts and the median follow-up time of patients still alive at the time of the analysis was 88 months.

**Table 1** Effect of tumour cathepsin D on relative relapse and death rates

| Cathepsin D status | Relative relapse rate | Relative death rate |
|---|---|---|
| Q2 vs Q1 | 1.27 (1.08–1.50) | 1.36 (1.13–1.64) |
| Q3 vs Q1 | 1.45 (1.24–1.71) | 1.52 (1.26–1.82) |
| Q4 vs Q1 | 1.48 (1.26–1.74) | 1.56 (1.31–1.87) |

Q1, 0–33; Q2, >33–47; Q3, 47–70; Q4, >70 pmol mg$^{-1}$ protein.

**Table 2** Effect of tumour cathepsin D on relapse-free and overall survival

| Cathepsin D status | RFS (% ± s.e.) | Median RFS (months) | OS (% ± s.e.) | Median OS (months) |
|---|---|---|---|---|
| Q1 | 55 ± 3 | 137 | 63 ± 2 | 156 |
| Q2 | 48 ± 2 | 108 | 56 ± 2 | 134 |
| Q3 | 42 ± 3 | 77 | 53 ± 2 | 127 |
| Q4 | 36 ± 3 | 65 | 43 ± 3 | 101 |

Q1, 0–33; Q2, >33–47; Q3, 47–70; Q4, >70 pmol mg$^{-1}$ protein. RFS, relapse-free survival. OS, overall survival.

*Received 24 June 1998*
*Accepted 26 June 1998*

The majority of the data were analysed by separating cathepsin D levels into quartiles or by dichotomizing the data using a cut-off value close to that of the median. Cathepsin D expression was not associated with grade but was associated with menopausal, node, oestrogen and progesterone receptor status as well as with age and tumour size. These associations were weak but were statistically significant because of the size of the study.

The major take-home message of the study of Foekens et al (1998) is that cytosolic cathepsin D levels are predictive of early relapse and survival not only in node-negative breast cancer patients but in all subgroups of breast cancer patients examined. This includes node-negative, node-positive, premenopausal and post-menopausal patients. Multivariate analysis established that the predictive value of cathepsin D was independent of the other markers of prognosis examined. Of great significance was the impact that cathepsin D expression was found to have on relapse-free and overall survival. By multivariate analysis, relative relapse rates increased up to 1.48 and relative death rates up to 1.56 when comparing women with high and low levels of cathepsin D expression (Table 1). Survival analysis after 10 years of follow-up corroborated the importance of cathepsin D measurement in predicting relapse-free and overall survival. This analysis showed that there was approximately a 20% difference in relapse-free and overall survival probability between patients with the lowest (bottom 25%) and highest (top 25%) expression of cathepsin D. Expressed in a different way, median relapse-free survival dropped from 137 to 65 months and median overall survival dropped from 156 to 101 months with increasing cathepsin D expression (Table 2).

Clinical features such as nodal status are used to stratify patients for treatment. Part of the attraction of cathepsin D as a prognostic marker would be its value in predicting outcome within groups of patients defined by nodal status. The use of cathepsin D to identify node-negative patients with a high risk of relapse would have considerable resource implications for treatment. Based on the figures in the Foekens study, 10% of all patients would be targeted for more aggressive therapy if node-negative women expressing the highest levels (fourth quartile) were given additional therapy. This figure would rise to 23% if all node-negative cases containing more than the median value of 47 pmol $mg^{-1}$ protein were included. There is, as yet, no information available on the actual benefit of such a strategy, and clinical trials would obviously be required to answer this question.

The clear demonstration that elevated cathepsin D expression is definitely associated with a poor prognosis raises some tantalizing biological issues, some of which are addressed in the existing cathepsin D literature. One issue that continues to resurface is that of the cellular localization of the cathepsin D in breast tumours. The original identification of cathepsin D as an oestrogen-regulated protein was made using malignant breast epithelial cells in tissue culture, and immunohistochemical studies have confirmed that malignant epithelial cells in tumours make cathepsin D. However, a number of immunohistochemical studies have questioned the assumption that it is the amount of cathepsin D in the tumour cells that is of prognostic value and suggested that it is expression in the stromal cells that is significant (O'Donaghue et al, 1995; Nadji et al, 1996). This issue is important in understanding the biological role of cathepsin D in breast tumours, because focusing on expression in stromal cells challenges the assumption that tumour cells are responsible for their own invasive potential. Another issue is that of secretion. Cathepsin D is secreted from breast cancer cell lines, and this has considerable mechanistic appeal because it could explain how a normally intracellular protease could play its part in facilitating invasion and metastasis. None of the current assays, however, is capable of specifically measuring extracellular cathepsin D and, perversely, immunohistochemical assays only assess intracellular cathepsin D levels. There may, of couse, be a relationship between the amount of intracellular and secreted cathepsin D, but it would be valuable to try to measure the amount of this protease in the extracellular space in tumours and to assess its prognostic value.

Finally, the question remains as to whether it is the proteolytic activity of cathepsin D that it responsible for the poor prognosis. It remains a possibility, for instance, that increased cathepsin D could reflect a general oversupply of lysosomal proteases and that some other protease is biologically more important. It has always been difficult to rationalize the importance of cathepsin D when it has such a low pH optimum, although it has been suggested that the pH of a poorly vascularized tumour may be sufficiently acidic to allow significant protease activity. Should cathepsin D become a contender as a clinically important prognostic marker guiding treatment, this issue would be highly relevant to oncologists wishing to make decisions about the treatment of women with high levels of cathepsin D in their tumours. If cathepsin D reflects a poor prognosis without being involved biologically, then more aggressive treatment could rely on the current repertoire of anti-cancer treatments. If, however, it transpires that cathepsin D is partially or wholly responsible for the aggressive behaviour of tumours, then the development of specific inhibitors of this enzyme would become increasingly urgent.

### REFERENCES

Benraad ThJ, Geurts-Moespot A, Sala M, Piffanelli A, Ross A and Foekens JA on behalf of the EORTC Receptor Study Group (1992) Quality control of cathespin D measurement by the EORTC Receptor Study Group. *Eur J Cancer* **28**: 72–75

Foekens JA, Berns EMJJ, Look MP and Klijn JGM (1996) Prognostic factors in node-negative breast cancer. In *Hormone-Dependent Cancer*, Pasqualini JR and Katzenellenbogen BS (eds), pp. 217–252, Marcel Dekker: New York

Foekens JA, Look MP, Bolt-deVries J, Meijer-van Gelder ME, van Putten WLJ and Klijn JGM (1998) Cathepsin D in primary breast cancer: prognostic evaluation involving 2810 patients. *Br J Cancer* **79**: 300–307

Nadji M, Fresno M, Nassiri M, Conner G, Herrero A and Morales AR (1996) Cathepsin D in host stromal cells, but not in tumour cells, is associated with aggressive behaviour in node-negative breast cancer. *Hum Pathol* **27**: 890–895

O'Donaghue AEMA, Poller DN, Bell JA, Galea MH, Elston CW, Blamey RW and Ellis IO (1995) Cathepsin D in primary breast carcinoma: adverse prognosis is associated with expression in cathepsin D in stromal cells. *Breast Cancer Res Treat* **33**: 137–145

Spyratos F, Brouillet JP, Defrenne A, Hacene K, Rouesse J, Maudelonde T, Brunet M, Andrieu C, Desplaces A and Rochefort H (1989) Cathepsin-D: an independent prognostic factor for metastasis of breast cancer. *Lancet* **i**: 1115–1118

Westley BR and May FEB (1996) Cathepsin D and breast cancer. *Eur J Cancer* **32**A: 15–24

Westley BR and Rochefort H (1980) A secreted glycoprotein induced by estrogen in human breast cancer cell lines. *Cell* **20**: 353–362

© Cancer Research Campaign 1999

